# Multifunctional injectable GelMA hydrogel incorporating Icariin-loaded Mesoporous silica nanoparticles to promote osseointegration

**DOI:** 10.1016/j.isci.2025.114061

**Published:** 2025-11-15

**Authors:** Yingjie Zhu, Chenyi Zhu, Yanfeng Tang, Yudong Jia, Youwen Liu, XianTao Chen, Chaowei Guo, Hongjun Li, Yuankun Zhai, Lihong Fan

**Affiliations:** 1 Xi’an Jiaotong University, Xi’an 710004, P.R. China; 2 Medical Center of Hip, Luoyang Orthopedic-Traumatological Hospital, Luoyang 471000, P.R. China; 3 School of Stomatology HENU, Kaifeng 475000, P.R. China

**Keywords:** Molecular biology, Bioengineering, Cell biology

## Abstract

The repair of bone defects faces the dual challenges of limited autograft availability and the single functionality of artificial materials. Consequently, the development of new biomaterials with mechanical adaptability, controlled drug release, and osteoinductive properties has become a major research focus in bone tissue engineering. In this study, we designed an injectable, photocrosslinkable hydrogel by incorporating icariin (ICA)-loaded mesoporous silica nanoparticles (MSNs) into gelatin methacryloyl (GelMA). The MSNs increased the compressive modulus by 1.5-fold and enabled pH-responsive ICA release. *In vitro* experiments demonstrated that the composite hydrogel enhanced rat bone marrow mesenchymal stem cell (rBMSC) proliferation and exhibited superior osteogenic differentiation capacity. *In vivo*, micro-CT analysis revealed a significantly higher bone volume fraction (BV/TV) in rat calvarial defects at 12 weeks, while histological examination confirmed the formation of mature trabecular bone. Overall, this MSN-mediated spatiotemporal delivery system effectively integrates immunomodulation with osteogenesis, providing a promising strategy for non-load-bearing osseointegration.

## Highlights

• A bespoke method quantifies multi-dimensional behavioral plasticity as behavioral entropy
• Clonal fish showed substantial arcs of plasticity through development
• This plasticity first increased for two weeks, then decreased
• Demonstrates the value of long-term tracking for testing developmental theory

## Graphical abstract

**Figure:**
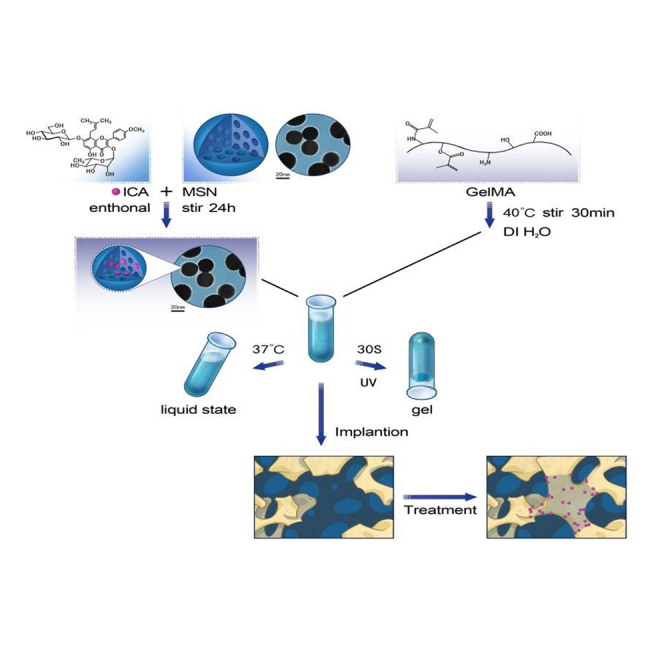

## Introduction

The high incidence of bone defects and their burden on the global public health system have become major challenges in the field of orthopedics. Each year, more than 2 million cases of critical bone defects arise from trauma, infection, or tumor resection.^1^ Despite being considered the gold standard, traditional autologous bone transplantation presents several limitations, including donor scarcity, secondary surgical injury, immune rejection, and poor shape matching.^2^ To address these issues, synthetic biomaterials such as hydroxyapatite and poly (lactic-co-glycolic acid) (PLGA) scaffolds have been investigated as alternatives. Although these substitutes partially alleviate donor shortages, their insufficient mechanical stability (compressive modulus < 5 kPa) and limited biological activity often result in suboptimal osseointegration.^3^^,^^4^ Therefore, the development of bone repair materials with improved mechanical adaptability, enhanced bioactivity, and controllable degradation has become an urgent priority in tissue engineering.

Gelatin methacryloyl (GelMA) hydrogel is a gelatin-derived material modified with methacrylic anhydride. In recent years, it has been widely applied in bone regeneration research due to its excellent biocompatibility, tunable light-induced polymerization, and biomimetic extracellular matrix (ECM)-like structure.^5^ At physiological temperature, GelMA remains liquid, but under ultraviolet (UV) irradiation, it undergoes polymerization and crosslinking to form a three-dimensional (3D) porous network. This process enables minimally invasive injection, *in situ* molding, and customized filling of complex bone defects.^6^^,^^7^^,^^8^ Despite these advantages, GelMA suffers from inherent drawbacks, including insufficient mechanical strength (typically <20 kPa) and rapid drug release (often >60% within 48 h), which severely restrict its application in load-bearing bone repair.^9^ Moreover, GelMA lacks intrinsic osteoinductive capacity and therefore depends on the incorporation of exogenous growth factors, such as BMP-2, to promote bone regeneration.^10^ To overcome these limitations, researchers have attempted to introduce inorganic nanoparticles (e.g., hydroxyapatite and silica) or growth factors to improve mechanical properties and impart bioactivity. However, these strategies face challenges such as nanoparticle agglomeration, the high cost of growth factors, and instability of their biological activity.^11^

To address the above limitations, researchers have sought to optimize the physicochemical and biological properties of GelMA hydrogels through composite material strategies. Mesoporous silica nanoparticles (MSNs) possess a high specific surface area (>1000 m^2^/g), tunable pore sizes (2–10 nm), and facile surface functionalization, making them suitable both as drug carriers and as mechanical reinforcement units.^12^^,^^13^^,^^14^ Previous studies have demonstrated that MSNs can enhance hydrogel mechanical properties through nanoparticle crosslinking, while their mesoporous channels enable efficient loading and sustained release of proteins, genes, or small-molecule drugs.^15^ Furthermore, the ordered pore structure of MSNs allows pH-responsive, controlled drug release, accelerating release under the mildly acidic conditions of bone defects (pH ∼6.5), which aligns with the requirements for osteogenic repair during the late inflammatory stage.^16^ In addition, the silicic acid ions (Si^4+^) generated during MSN degradation can activate osteogenesis-related genes such as Runx2 and Osterix, thereby promoting the osteogenic differentiation of BMSCs.^17^ Bioactive silica-based nanoparticles have also been reported to stimulate osteoblast differentiation and mineralization.^18^ Although MSNs have been incorporated into synthetic polymers such as PLGA for bone repair,^19^ their dispersion stability and synergistic biofunctionality with natural hydrogels like GelMA remain insufficiently explored.

To improve the bioavailability of hydrogels, bioactive substances have been incorporated into their structure. Bone morphogenetic protein-2 (BMP-2) is a potent growth factor known to induce stem cell differentiation and promote both bone and cartilage formation. However, its high cost and the potential life-threatening risks associated with supraphysiological concentrations represent significant drawbacks.^20^ Icariin (ICA), the principal active component of the traditional Chinese medicinal herb *Epimedium*, exhibits strong osteogenic and angiogenic activities. Mechanistic studies have shown that ICA upregulates osteogenic markers such as alkaline phosphatase (ALP) and osteocalcin (OCN) by activating the cAMP and Wnt/β-catenin signaling pathways.^21^^,^^22^ Additionally, ICA promotes the secretion of vascular endothelial growth factor (VEGF), thereby achieving an “osteogenesis-angiogenesis” coupling effect.^23^ Compared with recombinant growth factors, ICA offers the advantages of low cost, high stability, and minimal immunogenicity.

**Figure 1:**
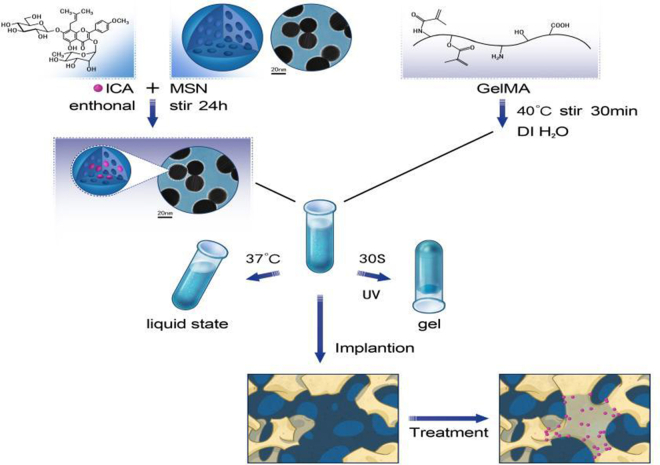
Schematic diagram of the composite scaffold for bone defect repair

The innovation of this study lies in the development of a GelMA/MSN/ICA composite hydrogel system with injectability, photoresponsive crosslinking, and dual functionalization. The preparation process is illustrated in Figure 1. By covalently integrating ICA-loaded MSNs into the GelMA network, we constructed a pH-responsive drug reservoir while simultaneously enhancing gel stiffness. This system enables stepwise ICA release, an initial burst to suppress inflammation followed by sustained release to promote mineralization, while MSN-mediated mechanical cues synergistically regulate the osteogenic differentiation of rat bone marrow mesenchymal stem cells (rBMSCs). The objectives of this study were to systematically characterize the physicochemical properties of the hydrogel, evaluate its osteogenic differentiation capacity both *in vitro* and *in vivo*, and assess its bone defect repair efficiency in a rat calvarial defect model. Collectively, these investigations aim to verify the bone regeneration potential of the composite hydrogel and provide a novel strategy for the design of multifunctional scaffolds for bone repair.

## Results

### Microstructural characterization of the composite hydrogel

**Figure 2:**
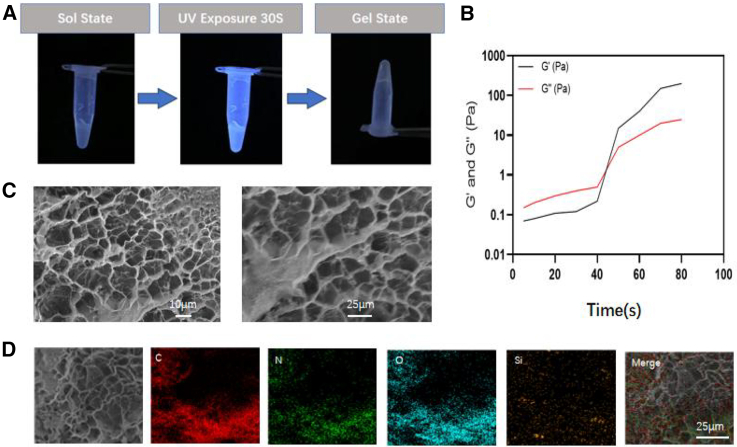
Characterization of composite scaffold (A) Appearance of the GelMA/MSN/ICA hydrogel. Composite hydrogel is sol state at body temperature and turned to gel state after UV photocrosslinking. (B) Rheological testing of GelMA/MSN/ICA hydrogel. (C) Microstructure of GelMA/MSN/ICA hydrogel. (D) EDS-SEM of GelMA/MSN/ICA hydrogel. All values are expressed as mean ± SD, *n* = 3.

As shown in Figure 2A, the GelMA/MSN/ICA hydrogel was successfully prepared and observed *in vitro*. The composite hydrogel was injectable at body temperature and underwent rapid gelation after UV-induced photocrosslinking. Rheological analysis (Figure 2B) revealed that the storage modulus (*G*″) was initially higher than the loss modulus (*G*′), indicating a sol state. Upon UV irradiation, *G*′ increased sharply and eventually surpassed *G*″, with a crossover point demonstrating a clear sol-gel phase transition. SEM imaging (Figure 2C) showed that the GelMA/MSN/ICA hydrogel exhibited a well-defined interconnected porous structure with pore sizes ranging from 5 to 20 μm. The MSNs were uniformly distributed within the GelMA matrix. Furthermore, EDS element mapping (Figure 2D) confirmed the presence of C, N, O, and Si, validating the uniform incorporation of MSNs and collagen components throughout the hydrogel. Mechanical testing demonstrated that the GelMA/MSN/ICA hydrogel maintained its irregular pore geometry and overall structural integrity under compression. Importantly, the compression modulus of the composite hydrogel (12.3 ± 1.2 kPa) was significantly higher than that of the pure GelMA hydrogel (8.1 ± 0.9 kPa, ∗∗*p* < 0.05), representing a ∼1.5-fold enhancement. This improvement in mechanical strength suggests that MSNs incorporation not only reinforces hydrogel stability but may also provide a more favorable microenvironment for cell adhesion and subsequent osteogenic activity.

### Swelling and degradation behavior of the composite hydrogel

**Figure 3:**
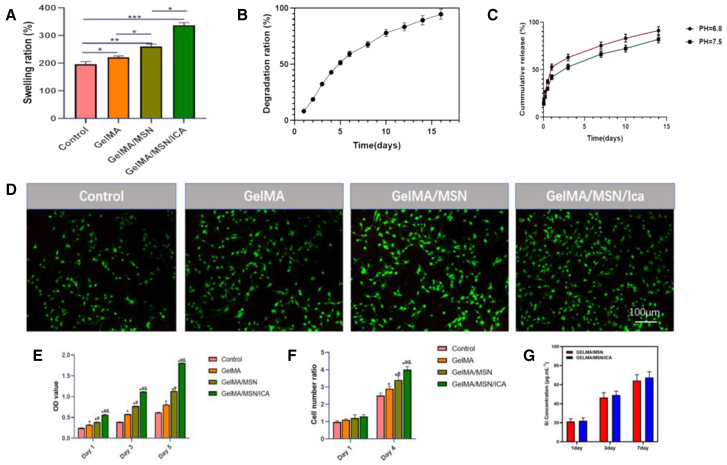
Material analysis and cell experiment (A) Swelling ratio of GelMA/MSN/ICA hydrogel. (B) Degradation rate of GelMA/MSN/ICA hydrogel. (C) Release rate of ICA in the composite system in different pH conditions (pH – 6.8, pH – 7.5). (D)Live/dead staining of rBMSCs on 4 days (living cells were green and dead cells were red; bars, 1 mm). (E) Proliferation of rBMSCs on 1, 3, and 5 days evaluated using CCK-8. (F) Cell number ratio of BMSCs cultured with the control group, GelMA, GelMA/MSN, and GelMA/MSN/ICA group for 1 and 4 days, respectively. (G) The release of Si ions from GelMA/MSN and GelMA/MSN/ICA in Dulbecco’s modified eagle medium (DMEM) at 37°C for 1, 3, and 7 days (*p* > 0.05). All values are expressed as mean ± SD (*n* = 3, ∗indicates significant differences between groups, ∗*p* < 0.05; ∗∗*p* < 0.01; ∗∗∗*p* < 0.001).

After immersion in PBS for 24 h, the equilibrium swelling ratio of the GelMA/MSN/ICA hydrogel (336.8 ± 9.6%) was significantly higher than that of the GelMA hydrogel (220.7 ± 5.6%) (Figure 3A). The incorporation of MSNs may help protect the GelMA network from excessive hydration, balancing hydrophilicity with structural integrity. This enhanced swelling capacity suggests an improved potential for nutrient exchange under dynamic physiological conditions. *In vitro* degradation analysis demonstrated that the GelMA/MSN/ICA hydrogel gradually degraded over 16 days, reaching a degradation rate of 94.5 ± 4.5% by day 16 (Figure 3B), indicating favorable biodegradability. The MSNs component may stabilize GelMA against enzymatic hydrolysis by collagenase, allowing controlled and sustained degradation that aligns with the bone repair process. Regarding the release effect of silicate ions (Si^4+^) in GelMA/MSN/ICA and GelMA/MSN, it was observed that the Si content in each group increased with time, reaching approximately 40% after 7 days of immersion. There were no significant differences between the two groups at various time points (Figure 3G).

### ICA release rate

The release profile of ICA from the GelMA/MSN/ICA hydrogel was monitored over 15 days (Figure 3C). An initial burst release was observed, with 52.7 ± 3.0% of ICA released on the first day. Following this phase, the release rate gradually slowed and stabilized, maintaining a sustained release for the remainder of the 15-day period. By day 15, the cumulative release reached 91.2 ± 4.2%, demonstrating that the MSN mesoporous channels effectively captured ICA molecules and regulated their diffusion. Moreover, the composite hydrogel was found to generate mildly acidic conditions (pH ∼6.8). When the microenvironment was adjusted to a weakly alkaline condition (pH ∼7.5) and ICA release was measured, both groups showed a continuous and significant increase in cumulative release over time. However, the release rate was consistently and significantly higher at pH 6.8 than at pH 7.5, indicating that ICA release exhibited pH responsiveness. This sustained release behavior of the composite hydrogel is an important feature for supporting long-term osteogenesis. Notably, the initial pH and enzyme-responsive release curve aligned with the temporal requirements of bone regeneration, from the early inflammatory phase to the later remodeling stage.

### Cell morphology assessment

**Figure 4:**
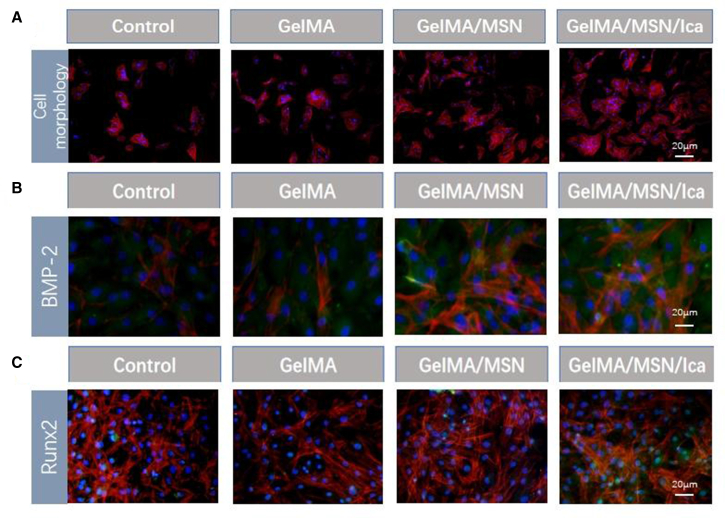
Cell adhesion test and protein staining (A) Morphology of rBMSCs on day 1 (cytoskeleton was stained with TRITC-phalloidin [red] and nuclei was stained with DAPI [blue]). (B) Morphology of rBMSCs on 7 days, and the protein staining of BMP-2 (Green). (C) Morphology of rBMSCs on 7 days, and the protein staining of Runx2 (Green).

The effect of the composite hydrogel on rBMSC morphology was evaluated using phalloidin/4ʹ,6-diamidino-2-phenylindole (DAPI) staining (Figure 4A). In all groups, including blank control, GelMA, GelMA/MSN, and GelMA/MSN/ICA, rBMSCs displayed well-spread and intact morphology, with phalloidin staining revealing distinct and organized cytoskeletal filaments. Immunofluorescence staining of Runx2 and BMP-2 (Figures 4B and 4C) showed green fluorescence corresponding to protein expression. Notably, rBMSCs cultured on GelMA/MSN/ICA hydrogel exhibited higher Runx2 and BMP-2 expression compared with the other groups, while maintaining normal cell morphology. These results indicate that the composite hydrogel does not negatively affect early cell adhesion and effectively supports osteogenic protein expression in rBMSCs.

### Cell viability assessment

Cell viability and proliferation were assessed using CCK-8 assay and live/dead staining. The CCK-8 results showed that by day 5, the optical density (OD) of the GelMA/MSN/ICA group was 1.43 ± 0.04 times higher than that of the GelMA group (Figure 3E), indicating significantly enhanced cell proliferation. Live/dead staining (Figures 3D and 3F) revealed that rBMSCs in the GelMA/MSN/ICA group were densely populated, gradually covering the entire field of view, with strong green fluorescence (calcein AM) and minimal red signal, confirming high cell viability. The improved cell proliferation is likely due to the synergistic effects of MSN nano-morphology, which provides structural support, and ICA, which exerts mitogenic activity. Together, these factors create a favorable microenvironment that promotes rBMSC expansion.

### Osteogenic differentiation *in vitro*

**Figure 5:**
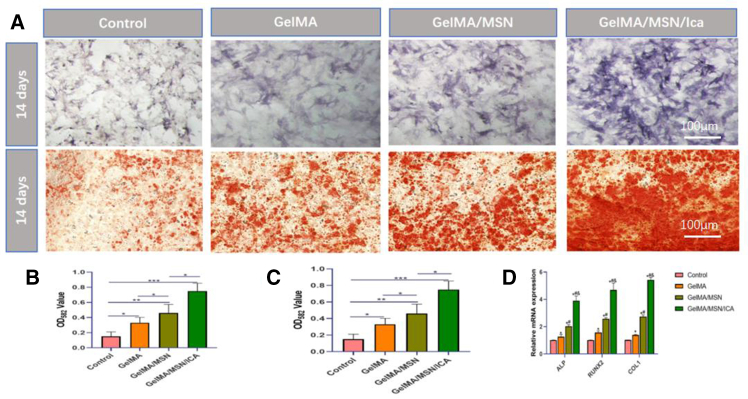
Osteogenic staining test (A) Evaluation of rBMSC differentiation using alkaline phosphatase staining and Alizarin red staining. (B) ALP activity of rBMSCs on 7 days. (C) Quantification of ECM mineralization on 14 days. (D) Relative fold change of osteogenic gene (ALP, Runx2, and Col1) expression measured by RT-qPCR on 7 days. All values are expressed as mean ± SD, *n* = 3.

Osteoblasts, which originate from the differentiation of rBMSCs, are the primary functional cells involved in bone regeneration. To evaluate osteogenic differentiation, rBMSCs cultured on hydrogels for 7 and 14 days were assessed using ALP and ARS staining (Figure 5A). ALP, an early osteogenic marker, showed pronounced staining in the GelMA/MSN/ICA group on day 7. Quantitative analysis revealed that ALP activity in the GelMA/MSN/ICA group was 1.41 ± 0.05 times higher than in the GelMA/MSN group and 2.92 ± 0.15 times higher than in the GelMA group (Figure 5B). By day 14, ARS staining demonstrated extensive calcium nodule formation in the GelMA/MSN/ICA group (Figure 5C), indicating effective mineralization and the progression of early differentiation markers into ECM deposition. These results collectively suggest that the GelMA/MSN/ICA hydrogel strongly promotes the osteogenic differentiation of rBMSCs.

### RT-qPCR analysis

RT-qPCR was employed to assess the expression of osteogenic differentiation-related genes in rBMSCs, including ALP, Runx2, and Col1, to evaluate the effect of the composite hydrogel (Table 1). On day 7, ALP expression in the GelMA/MSN/ICA group was 1.91 ± 0.04 times higher than that in the GelMA/MSN group and 2.87 ± 0.08 times higher than in the GelMA group. Similarly, Runx2 and Col1 were markedly upregulated in the GelMA/MSN/ICA group compared with the other groups (Figure 5D). Overall, these RT-qPCR results indicate that the GelMA/MSN/ICA hydrogel significantly enhances osteogenic differentiation of rBMSCs. This effect may be attributed to the sustained release of ICA and the synergistic activation of osteogenic signaling pathways, including BMP-2/Smad and Wnt/β-Catenin, mediated by MSN-derived silicic acid.

**Table 1:** Primers used in RT-qPCR

Gene	Forward Primer	Reverse Primer
Alp	GCTAGTGGAAGGAGGCAGGA	ATCCATCTCCACGGCCTCAT
RUNX2	ATCCAGCCACCTTCACTTACACC	GGGACCATTGGGAACTGATAGG
Col1	TGTTGGTCCTGCTGGCAAGAATG	GTCACCTTGTTCGCCTGTCTCAC

### Animal experimental evaluation

**Figure 6:**
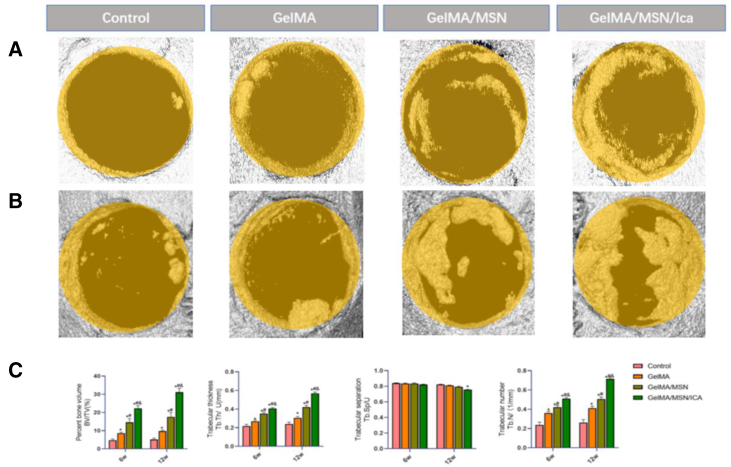
Micro CT imaging and analysis (A) Micro-CT images of the skull bone defect at 6 weeks. (B) Micro-CT images of the skull bone defect at 12 weeks. (C) Micro-CT analysis of bone regeneration with the control group, GelMA, GelMA/MSN, and GelMA/MSN/ICA group at 6 and 12 weeks (*n* = 3). All values are expressed as mean ± SD, *n* = 3.

Throughout the 6- to 12-week post-operative observation period, no infections or deaths were observed. Bone tissues, including the defect regions of comparable size, were randomly collected from each group for analysis. Bone ingrowth at the skull defect site was evaluated using micro-CT imaging (Figure 6A). At week 6, the GelMA/MSN/ICA group exhibited slightly greater bone regeneration compared with the GelMA/MSN group and significantly higher regeneration than the GelMA group. By week 12, continuous bone ingrowth was observed in all groups; however, the GelMA/MSN/ICA group showed the most pronounced bone regeneration, with differences between groups becoming more pronounced over time. Quantitative micro-CT analysis (Figure 6B) revealed that BV/TV, Tb.Th, and Tb.N in the GelMA/MSN/ICA group were significantly higher than that in the GelMA and GelMA/MSN groups at both 6 and 12 weeks, with the differences between groups increasing over time. These results indicate that the composite hydrogel effectively enhances bone regeneration *in vivo*.

**Figure 7:**
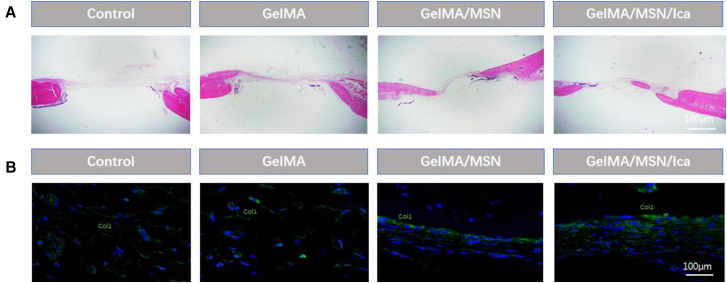
H&E staining and immunofluorescence staining (A) H&E staining of the skull defect at 12 weeks. (B) Immunofluorescence staining of Col1 (green). *n* = 3.

Hematoxylin and eosin (H&E) staining of the skull defects at 12 weeks revealed mature trabecular bone formation in the GelMA/MSN/ICA group, which was more pronounced than in the GelMA/MSN group, whereas the blank group exhibited prominent fibrous tissue infiltration (Figure 7A). Immunofluorescence staining of decalcified bone sections was performed to assess local bone regeneration, focusing on the osteogenic marker Col1 (Figure 7B). The GelMA/MSN/ICA group showed markedly higher fluorescence intensity compared with the other groups, indicating enhanced Col1 expression and superior bone regeneration.

## Discussion

Bone defects resulting from trauma, tumor resection, or congenital malformations remain a major clinical challenge, with millions of patients worldwide requiring treatment each year. Traditional approaches, including autologous and allogeneic transplantation, are limited by donor site availability, immune rejection, and insufficient mechanical stability. In contrast, synthetic materials such as ceramics and metals often lack biological activity and fail to replicate the dynamic bone remodeling microenvironment.^24^^,^^25^^,^^26^ GelMA is a photocrosslinkable hydrogel derived from the ECM. Its tunable physicochemical properties, excellent biocompatibility, and capacity to support osteogenic differentiation make GelMA a promising candidate for bone tissue engineering.^27^^,^^28^ However, GelMA-based scaffolds typically exhibit suboptimal mechanical strength, rapid degradation, and limited osteoinductive potential, which restrict their clinical application for critical-sized bone defects.^29^ To address these limitations, we developed a composite hydrogel composed of GelMA, MSNs, and ICA. This system provides a stable microenvironment for cell proliferation and differentiation, controlled and sustained degradation, appropriate mechanical properties, and efficient drug release, collectively enhancing bone regeneration and offering a promising solution for bone tissue engineering applications.

Our GelMA/MSN/ICA composite hydrogel addresses these limitations through the rational integration of MSNs and ICA. Human cancellous bone exhibits a porosity of 75%–90% and pore diameters ranging from approximately 50–300 μm.^30^ GelMA hydrogels can be freeze-dried to create porous scaffolds with controllable pore size and porosity.^31^ For example, Chen et al.^32^ synthesized GelMA hydrogels with different degrees of substitution (49.8%, 63.8%, and 73.2%) using 1, 5, and 10 mM solutions, respectively. The resulting freeze-dried scaffolds had average pore sizes of 50 μm (49.8%), 30 μm (63.8%), and 25 μm (73.2%) as characterized by SEM. These values indicate that the pore structure of our composite hydrogel closely matches the physiological parameters of human bone, providing a favorable microenvironment for nutrient exchange.^22^ Additionally, Liu et al. reported that MSN-doped polycaprolactone scaffolds increased compressive strength by 40% while enabling sustained drug release.^33^ Consistently, our rheological tests demonstrated that the composite hydrogel exhibits excellent injectability and rapidly transitions to a gel state under UV photocrosslinking. The swelling ratio further indicated that the inclusion of MSNs enhances crosslinking density. EDS analysis confirmed the uniform distribution of Si ions throughout the hydrogel, which, together with ICA, contributes to synergistic osteoimmunomodulation. The evenly dispersed MSNs also produces a rough surface topology, enhancing mechanical strength while maintaining irregular pore geometry and structural integrity. This combination of well-defined porosity and MSN incorporation provides ample space for cell adhesion and nutrient transport, creating an optimal microenvironment for bone tissue regeneration.^34^

The degradation of the composite hydrogel stabilized after 16 days, and degradation was incomplete, likely because MSNs enhanced direct interactions between the hydrogel and cells. The residual composite hydrogel provided a localized structural support without impeding bone remodeling. Previous studies have shown that growth factors such as BMP-2 loaded into MSNs significantly promote the osteogenic differentiation of mesenchymal stem cells.^35^ Similarly, in this system, ICA is carried by MSNs, and the gradual degradation of the hydrogel facilitates sustained release of MSNs, thereby enabling continuous ICA delivery. The initial burst and subsequent sustained release of ICA are attributed to the mesoporous structure of MSNs, which physically adsorbs ICA and enables pH- and enzyme-responsive diffusion.^13^^,^^36^ The later-stage sustained release is likely associated with the slow hydrogel degradation, aligning with the phased requirements of bone repair. During early inflammation (pH ∼6.8), MSN pores expand to accelerate drug release, while gradual release during the remodeling stage maintains osteogenic stimulation. Notably, the initial burst release (52.7% within 24 h) may also help suppress early inflammation.^37^^,^^38^

In previous studies, GelMA has often been used as a 2D or 3D cell culture matrix due to its inherently high biocompatibility^39^ and its abundance of RGD sequences, which promote cell adhesion.^40^^,^^41^ Although studies incorporating graphene oxide into GelMA achieved enhanced mechanical properties, cytotoxicity was observed at high filler concentrations (>7 wt %).^42^ In this study, phalloidin/DAPI staining revealed that rBMSCs in all groups exhibited well-spread morphology with bright, orderly filaments, indicating that the addition of ICA and MSNs did not negatively affect cell adhesion. Furthermore, CCK-8 assays and live/dead staining demonstrated that the number of cells in the composite hydrogel group was significantly higher than in the GelMA and GelMA/MSN groups by day 4, with cells displaying robust growth, highlighting the excellent biocompatibility of the composite hydrogel. In contrast, rBMSC proliferation in the GelMA-only group was slower, likely due to the absence of MSNs and ICA. The slight decrease in cell proliferation at later time points may be attributed to limited space in the culture well and contact inhibition. Overall, these results indicate that the GelMA/MSN/ICA composite hydrogel provides a supportive microenvironment for rBMSCs and effectively promotes their proliferation.

ALP and ARS staining were used to assess osteogenic differentiation. On day 7, ALP staining and quantitative analysis revealed that the GelMA/MSN/ICA composite hydrogel significantly enhanced early osteogenic differentiation compared with GelMA and GelMA/MSN groups. By day 14, ARS staining showed that mineralized and calcified nodules were most abundant in the GelMA/MSN/ICA group, indicating that the sustained release of ICA effectively promoted osteogenic maturation within 14 days. RT-qPCR analysis of osteogenic genes, including ALP, Runx2, and Col1, further confirmed these findings. Gene expression in the composite hydrogel group was significantly higher than in the other groups on both day 7 and day 14, demonstrating its strong osteoinductive capacity. ALP is a key early marker of osteogenesis, reflecting the differentiation of rBMSCs into osteoblasts,^43^ while Col1, a major bone matrix component, facilitates osteoblast adhesion and differentiation and is critical for bone matrix synthesis and mineralization.^44^ Mechanistically, ICA released from the hydrogel activates the ER α-Wnt/β-catenin and BMP-2/Smad signaling pathways, consistent with its dual role in promoting osteoblast differentiation and inhibiting osteoclast activity.^45^^,^^46^ Additionally, sustained silicic acid release from MSN degradation synergistically enhances Wnt/β-catenin signaling alongside ICA.^47^^,^^48^ Overall, the GelMA/MSN/ICA composite hydrogel effectively promotes the osteogenic differentiation of rBMSCs *in vitro*.

Animal experiments demonstrated that following skull defect creation and hydrogel implantation, micro-CT analysis revealed substantial bone regeneration at the defect sites in the GelMA/MSN/ICA group at both 6 and 12 weeks. Histological evaluation using H&E staining confirmed these observations, showing well-formed mature trabecular bone in the GelMA/MSN/ICA group, consistent with the trends observed in micro-CT images. Furthermore, immunofluorescence staining for Col1 demonstrated elevated expression at the implant interface, aligning with the *in vitro* gene expression results. These findings indicate that Col1 contributed effectively to promoting bone regeneration *in vivo*. Although the residual silica nanoparticles (<5 wt % at 12 weeks) are unlikely to cause acute toxicity, long-term biodistribution studies are necessary, as previous reports indicate that silica can accumulate in the liver and spleen for over 6 months.^49^ Second, while the rat skull defect model provides a standardized platform for evaluation, it does not replicate the mechanical loading conditions of human load-bearing bones.^50^ Validation of dynamic compression performance in larger animal models, such as sheep tibia, is warranted. Third, although MSNs enables sustained ICA release, the initial burst within the first 24 h may require further surface modification to achieve more precise temporal control.

In this study, we incorporated MSNs loaded with ICA into GelMA hydrogel to create a composite system aimed at enhancing bone regeneration and osseointegration. The addition of MSNs improved the mechanical stability and functionality of the hydrogel. ICA, uniformly distributed within the composite hydrogel via MSNs, was released over a period exceeding 15 days. This sustained local release of ICA promoted the proliferation and osteogenic differentiation of rBMSCs, thereby facilitating early-stage osseointegration. Notably, this study demonstrates that mixing MSNs with ICA in GelMA hydrogel can significantly promote osseointegration, which addressing a critical gap in the development of natural polymer-based composite scaffolds.

### Limitations of the study

This study has several main limitations. First, it did not directly verify the amino functionalization modification of MSNs. Moreover, the presence and role of amino groups were indirectly inferred from EDS and swelling analyses. In the future, direct characterization techniques such as infrared spectroscopy (IR) and X-ray photoelectron spectroscopy (XPS) should be employed to accurately determine the functionalization status and degree of modification, which is essential for establishing a definitive correlation between amino group incorporation and enhancements in mechanical properties, drug release behaviors, and other functional outcomes. Second, histological analysis such as H&E staining at 12 weeks has been completed, and bone repair is a long-term process. Thus, investigations capturing dynamic tissue morphological changes during earlier stages of bone healing are necessary to better predict and evaluate long-term therapeutic effects. Future work should also focus on the long-term degradation behavior and biocompatibility of the material under various physiological conditions. Lastly, further research is warranted to explore the potential of this composite hydrogel as a drug delivery platform, particularly its effects on cellular behaviors. Expanding its application in scenarios such as infection control and tissue regeneration will help unlock the full potential of multifunctional hydrogels and provide a stronger experimental foundation for translational medical research.

## Resource availability

### Lead contact

Further information and requests for reagents should be directed to and will be fulfilled by the lead contact, Prof. Lihong Fan (drfan2140@xjtu.edu.cn).

### Materials availability

All reagents generated in this study are available from the lead contact with a completed materials transfer agreement.

### Data and code availability

• All data reported in this paper are available from the lead contact upon request.
• This paper does not report original code.
• Any additional information required to reanalyze the data reported in this paper is available from the lead contact upon request.

## Acknowledgments

This work was supported by Henan Province 2025 Science and Technology Development Plan (grant no. 252102310375) and special project for Scientific Research of Traditional Chinese Medicine in Henan Province (grant no. 2023ZY2121).

## Author contributions

Y.Z. and C.Z. contributed to the conception and design of the study; C.G., X.Z., Y.Z., Y.T., H.L., Y. J., and X.C. contributed to the data acquisition, interpretation, and analysis; Y.L. and L.F. contributed to the data interpretation and revision of the manuscript. All authors approved the submission of the paper.

## Declaration of interests

The authors declare that they have no known competing financial interests or personal relationships that could have appeared to influence the work reported in this paper.

## STAR★Methods

### Key resources table

**Table:** 

REAGENT or RESOURCE	SOURCE	IDENTIFIER
**Antibodies**		

Type 1 collagen (Col1) primary antibody	Abcam	Cat#ab21286; 1:200
Alexa Fluor 488 coupled secondary antibody	Invitrogen	1:500

**Biological samples**		

Wistar rats	N/A	N/A

**Chemicals, peptides, and recombinant proteins**		

Gelatin methacryloyl (GelMA)	Suzhou, China	efl-gm-60
Amine-functionalized mesoporous silica nanoparticles (MSNs)	Sigma-Aldrich	N/A
Icariin (ICA)	Sichuan, China	N/A
Phenyl-2,4,6-trimethylbenzoylphosphonate (LAP)	Sigma-Aldrich	N/A
Dimethyl sulfoxide (DMSO)	N/A	N/A
Ethanol	N/A	N/A
Deionized water (DI H2O)	N/A	N/A
Phosphate-buffered saline (PBS)	N/A	N/A
Fetal bovine serum (FBS)	Gibco, Grand Island, NY, USA	N/A
Penicillin/streptomycin	N/A	N/A
4% Paraformaldehyde	Solarbio, Beijing, China	Invitrogen D1306
Triton X-100	N/A	N/A
TRITC-Phalloidin	Solarbio, Beijing, China	N/A
4',6-diamidino-2-phenylindole (DAPI)	Solarbio, Beijing, China; Invitrogen	N/A
Rhodamine	N/A	N/A
BCIP/NBT staining reagent	Beyotime, Shanghai, China	N/A
Alkaline phosphatase (ALP) detection kit	Beyotime, Shanghai, China	N/A
Alizarin red staining (ARS) solution	Beyotime, Shanghai, China	N/A
10% Cetylpyridinium chloride	N/A	N/A
Trizol reagent	Invitrogen, Carlsbad, CA, USA	N/A
PrimeScript RT Kit	Takara, Japan	N/A
Q SYBR green Supermix	Bio-Rad, Hercules, CA, USA	N/A
3% Pentobarbital Sodium	N/A	N/A
Ethylenediamine tetraacetic acid (EDTA)	N/A	N/A
Mayer’s hematoxylin	Sigma	N/A
Eosin Y	Sigma	0.5% 95% ethanol solution
Citrate buffer (10 mM, pH 6.0)	N/A	N/A
Normal goat serum	Vector Laboratories	N/A
1% Bovine serum albumin (BSA)	N/A	N/A
Low-glycemic medium (L-DMEM)	Gibco, Grand Island, NY, USA	N/A
L-DMEM osteogenic medium	Cyagen, Santa Clara, CA, USA	N/A

**Critical commercial assays**		

Live/dead staining kit	Bestbio, Shanghai, China	N/A
Cell counting kit-8 (CCK-8)	Irvine, CA, USA	N/A

**Deposited data**		

Raw and analyzed data of this study	This paper	N/A

**Experimental models: Cell lines**		

Rat bone marrow mesenchymal stem cells (rBMSCs)	Isolated from 3-week-old Wistar rats	Wang et al.^24^

**Experimental models: Organisms/strains**		

Wistar rats	Experimental animal model	N/A

**Oligonucleotides**		

ALP forward primer: GCTAGTGGAAGGAGGCAGGA	This paper	N/A
ALP reverse primer: ATCCATCTCCACGGCCTCAT	This paper	N/A
RUNX2 forward primer: ATCCAGCCACCTTCACTTACACC	This paper	N/A
RUNX2 reverse primer: GGGACCATTGGGAACTGATAGG	This paper	N/A
Col1 forward primer: TGTTGGTCCTGCTGGCAAGAATG	This paper	N/A
Col1 reverse primer: GTCACCTTGTTCGCCTGTCTCAC	This paper	N/A

**Software and algorithms**		

Image J software	N/A	N/A
GraphPad software	GraphPad, Inc., La Jolla, CA, USA	N/A
QuantStudioTM 7 Flex real-time PCR system	Applied Biosystems, Carlsbad, CA, USA	N/A
CTvox	N/A	N/A
Multimodal 3D visualization software	N/A	N/A
NIS elements ar 5.21 software	N/A	N/A
SPSS software version 26.0	SPSS Inc., Chicago, USA	N/A
Primer Premier software	Premier Biosoft, Palo Alto, CA, USA	N/A

**Other**		

Field emission scanning electron microscopy (FE-SEM)	Hitachi su8010	N/A
Energy dispersive spectroscopy (EDS) with SEM	N/A	N/A
Rotational rheometer (TA instruments discovery HR-2)	TA instruments	N/A
Ultraviolet (UV) spectrophotometer (lambda 800)	PerkinElmer, USA	N/A
Fluorescence microscope (Olympus IX71)	Olympus, Tokyo, Japan	N/A
Bright field microscope (Nikon Eclipse Ni-E)	Nikon	N/A
Confocal microscope (Zeiss LSM 880 Airyscan)	Zeiss	N/A
Micro computed tomography (Micro-CT) system (Skyscan 1076 scanner)	Kontich, Belgium	N/A
Branson 5800 ultrasonic homogenizer	Branson	40KHz
OmniCure S2000 ultraviolet light source	N/A	365nm, 10mW/cm^2^
Bio-Rad microplate reader	Bio-Rad	N/A

### Method details

#### ICA loading into MSNs

Amine-functionalized MSNs (100 mg; Sigma-Aldrich) were uniformly dispersed in 0.5 mL of ethanol and mixed with 5 mg of ICA (Sichuan, China) dissolved in DMSO. The mixture was stirred at room temperature under dark conditions for 24 hours. Drug-loaded nanoparticles were then collected by centrifugation (12,000 rpm, 10 min), washed three times with ethanol, and dried at room temperature. The drug loading capacity (DLC) was calculated using the following equation: DLC (%) = (mass of loaded ICA/total mass of MSN@ICA) × 100% = 2.35 ± 0.12% (∗n∗ = 3).^51^

#### Synthesis and characterization of GelMA/MSN/ICA hydrogels

Freeze-dried GelMA (100 mg; efl-gm-60; Suzhou, China) was dissolved in 1.0 mL of deionized water (DI H_2_O) containing the photoinitiator phenyl-2,4,6-trimethylbenzoylphosphonate (LAP, 0.3% w/v; Sigma) at 50 °C in the dark. The prepared MSN/ICA (0.25% w/v) was then added, and the mixture was homogenized by ultrasonic treatment for 15 min (40 kHz; Branson 5800) to ensure uniform dispersion. The precursor solution (200 μL) was transferred into test tubes and photocrosslinked under UV light (365 nm, 10 mW/cm^2^; OmniCure S2000) for 30 s. The microstructure of the resulting hydrogel was examined by field-emission scanning electron microscopy (FE-SEM; Hitachi SU8010). The distribution of MSN nanoparticles within the hydrogel was analyzed by surface scanning using energy-dispersive spectroscopy (EDS) coupled with SEM.

#### Rheological and swelling characterization

The rheological properties of the hydrogels were analyzed at 37 °C using a rotational rheometer (Discovery HR-2; TA Instruments) equipped with a 20-mm parallel plate geometry. The storage modulus (G′) and loss modulus (G′′) were recorded under oscillatory shear conditions (frequency: 1.0 Hz; strain: 1%). Gelation time was defined as the point at which G′ reached 90% of its equilibrium modulus. The swelling behavior of the composite hydrogel was also evaluated. Pre-gel solution was injected into a mold, photocrosslinked under UV light for 30 s, dried, and weighed (W_0_). The hydrogel was then immersed in PBS for 24 h, subsequently dried, and weighed again (W_s_). The swelling ratio was calculated as: (W_s_ − W_0_)/W_0_ × 100%.

#### Degradation rate of hydrogel and measurement of Si^4+^

Degradation measurements were conducted on days 1, 2, 3, 4, 5, 6, 8, 10, 12, 14, and 16. At each time point, the residual hydrogels were collected and weighed. The degradation rate (R) was calculated based on weight changes using the following equation: R (%) = 100 × (W_2_-W_1_)/W_1_, where W_1_ represents the initial weight of the hydrogel and W2 represents the weight after degradation. In addition, qualitative degradation was assessed by rhodamine staining, with the extent of color fading used as an indicator of hydrogel breakdown. The degradation experiment *in vitro* was performed by immersing GelMA/MSN and GelMA/MSN/ICA in DMEM at 37°Con a shaker for 1, 3 and 7 days. The supernatant was collected at each time point and fresh DMEM was added back. The concentrations of free silicon were determined by inductively coupled plasma atomic emission spectrometer (ICP-AES, Thermofisher X series 2, USA).

#### ICA release rate

To evaluate the release profile of ICA from the composite hydrogel, samples were immersed in a 48-well culture plate containing 1 mL of phosphate-buffered saline (PBS) supplemented with fetal bovine serum (FBS) and maintained at 37 °C. The PBS was refreshed at predetermined intervals (1 h, 5 h, 12 h, 1, 3, 7, 10, 14, and 16 days). At each time point, the concentration of ICA in the release medium was determined by measuring absorbance at 270 nm using a UV spectrophotometer (Lambda 800; PerkinElmer, USA). The total ICA content in the composite hydrogel was quantified as 5× 10^-4^ μ mol. All experiments were performed independently in triplicate.

### Experimental model and study participant details

#### rBMSCs isolation and culture

rBMSCs were obtained from the bone marrow cavity of 3-week-old Wistar rats following a previously described protocol.^52^ The cells were cultured in low-glucose medium (Gibco, Grand Island, NY, USA) supplemented with 10% FBS and 1% penicillin/streptomycin under standard conditions (37 °C, 5% CO_2_, and 95% relative humidity). The medium was refreshed every 3 days. Cell growth and density were monitored microscopically, and when confluence reached approximately 90%, cells were passaged at a ratio of 1:3. Third-passage rBMSCs were used for cell viability and osteogenic differentiation assays. The experimental groups were as follows: (1) blank control, (2) GelMA, (3) GelMA/MSN, and (4) GelMA/MSN/ICA.

#### Cell morphology

The morphology of rBMSCs on the hydrogels was examined using cytoskeletal and nuclear staining. GelMA, GelMA/MSN, and GelMA/MSN/ICA hydrogels were polymerized under UV light for 30 s in a 48-well plate. A blank control group without any material was also included. Each group was seeded with an rBMSCs suspension (1 × 10^4^ cells/well) and incubated under standard conditions (37 °C, 5% CO_2_) for 24 h. After incubation, the samples were washed twice with PBS, fixed with 4% paraformaldehyde (Solarbio, Beijing, China) for 30 min, and permeabilized with 0.2% (V/V) Triton X-100. Cytoskeletal actin filaments were stained with TRITC-Phalloidin, and nuclei were counterstained with DAPI according to the manufacturer’s instructions (Solarbio, Beijing, China). Stained samples were imaged using a fluorescence microscope (Olympus IX71, Tokyo, Japan), with cytoskeletons visualized in red and nuclei in blue. Additionally, after 7 days of culture, BMP-2 and Runx2 expression in the hydrogels was detected by immunofluorescence staining. Cells were counterstained with phalloidin and DAPI, and fluorescence microscopy was used to observe protein expression.

#### Cell viability

The survival rate of rBMSCs in the hydrogels was evaluated using live/dead staining (BestBio, Shanghai, China) and a Cell Counting Kit-8 (CCK-8; Irvine, CA, USA). GelMA, GelMA/MSN, and GelMA/MSN/ICA hydrogels were polymerized under UV light for 30 s in 48-well plates. A blank control group without any material was also included. rBMSCs were seeded at a density of 1 × 10^4^ cells/well and cultured for 1 and 4 days. Live/dead staining was performed according to the manufacturer’s instructions, and samples were observed under a fluorescence microscope. Live cells stained with calcein-AM emitted green fluorescence, whereas dead cells stained with propidium iodide (PI) emitted red fluorescence. ImageJ software was used to quantify the ratio of live to dead cells. Cell viability was further assessed using the CCK-8 assay. rBMSCs cultured with the hydrogels for 1, 3, and 5 days were incubated with CCK-8 solution, and absorbance was measured at 450 nm using a microplate reader (Bio-Rad, USA). The cell survival rate (%) was calculated as: Cell Survival rate (%) = (test od blank OD)/(control od blank OD) × 100%. All experiments were performed in triplicate.

#### Osteogenic differentiation of rBMSCs in *vitro*

For osteogenic differentiation, the blank control, GelMA, GelMA/MSN, and GelMA/MSN/ICA hydrogels were polymerized under UV light for 30 s in 48-well plates. rBMSCs were seeded at a density of 5 × 10^4^ cells/well and cultured for 7 or 14 days in L-DMEM osteogenic medium (Cyagen, Santa Clara, CA, USA). On day 7, ALP expression was assessed using BCIP/NBT staining (Beyotime, Shanghai, China), and ALP activity was quantified with an ALP detection kit (Beyotime, Shanghai, China) according to the manufacturer’s protocol. On day 14, calcium deposition was evaluated using Alizarin red s (ARS) staining (Beyotime, Shanghai, China). At the designated time points, cells were washed twice with PBS, fixed with 4% paraformaldehyde (Solarbio, Beijing, China) for 30 min, and then incubated with the respective dye at room temperature for 30 min. Samples were subsequently washed twice with PBS to remove background staining. The stained samples were examined under a light microscope to observe ALP activity and calcium nodule formation. For quantitative analysis of calcium deposition, ARS-stained samples were decolorized with 10% cetylpyridinium chloride, and absorbance was measured at 562 nm using a UV spectrophotometer.

#### Reverse transcription quantitative polymerase chain reaction (RT-qPCR)

To evaluate the osteogenic differentiation potential induced by the composite hydrogel, the expression levels of osteogenesis-related genes, including ALP, runt-related transcription factor 2 (Runx2), and collagen type I (Col1), were analyzed by RT-qPCR. The blank control, GelMA, GelMA/MSN, and GelMA/MSN/ICA hydrogels were polymerized under UV light for 30 s in 48-well plates containing osteogenic medium. rBMSCs were seeded at a density of 1 × 10^5^ cells/well and cultured for 7 days. Total RNA was extracted using TRIzol reagent (Invitrogen, Carlsbad, CA, USA) and reverse-transcribed into cDNA with the PrimeScript RT kit (Takara, Japan). RT-qPCR was performed using iTaq™ SYBR Green Supermix (Bio-Rad, Hercules, CA, USA) on a QuantStudio™ 7 Flex Real-Time PCR System (Applied Biosystems, Carlsbad, CA, USA). GAPDH served as the internal control, and relative gene expression levels were calculated using the 2ˆ−ΔΔCt method. Data analysis was carried out using GraphPad Prism software (GraphPad Software Inc., La Jolla, CA, USA). All experiments were independently repeated three times. Primer sequences were designed using Primer Premier software (Premier Biosoft, Palo Alto, CA, USA) and are listed in Table 1.

#### Animal experiments

All animal procedures were conducted in strict accordance with the ethical guidelines of the Henan University School of Stomatology and were approved by the Animal Ethics Committee of Henan University School of Stomatology (HUSOM2025-764). A total of 40 healthy Wistar rats were randomly assigned to four groups (n = 10 per group) using a computer-generated randomization table: (1) blank control group; (2) GelMA group; (3) GelMA/MSN group; and (4) GelMA/MSN/ICA group. Rats were anesthetized by intraperitoneal injection of 3% pentobarbital sodium. Following anesthesia, the scalp was shaved and disinfected, and an incision was made to expose the calvarium. A standardized 5 mm × 5 mm critical-sized defect was created using a 5 mm trephine drill, and the periosteum at the defect margin was carefully removed to prevent spontaneous ossification. After thorough irrigation with sterile saline, the corresponding hydrogel solution was applied into the defect site and allowed to polymerize for 30 s under UV light. The incision was then sutured to complete model establishment. To prevent postoperative infection, penicillin (1.5 mg/kg) was administered intramuscularly three times daily for 3 consecutive days. At 6 and 12 weeks post-implantation, rats were euthanized by intravenous injection of an overdose of pentobarbital sodium, and the defect sites were harvested and fixed in 4% paraformaldehyde for further analysis.

#### Micro-CT analysis

To evaluate bone regeneration at the defect sites, micro-computed tomography (Micro-CT) was performed using a Skyscan 1076 scanner (Kontich, Belgium). Image datasets were reconstructed into 3D models using CTvox and multimodal 3D visualization software. Quantitative analysis was conducted to assess key parameters, including bone volume fraction (BV/TV), trabecular thickness (TB. Th), trabecular number (TB. N), and trabecular separation (TB. SP). Following Micro-CT scanning, the samples were decalcified in 10% ethylenediaminetetraacetic acid (EDTA) solution for 8 weeks, dehydrated through a graded ethanol series, and subsequently embedded in paraffin for histological analysis.

#### Hematoxylin-eosin(HE)staining

After decalcification and paraffin embedding, bone tissue specimens from each group were randomly selected for histological analysis. Sections were cut, dewaxed in xylene, and rehydrated through a graded ethanol series (100%, 95%, 80%, and 70%, 5 minutes each), followed by rinsing in distilled water. Nuclear staining was performed by immersing the slides in Mayer’s hematoxylin (Sigma, MH532) for 8 minutes, rinsing for 5 minutes, and differentiating in 1% acid ethanol (1% HCl in 70% ethanol) for 30 seconds. Cytoplasmic staining was carried out using Eosin Y (0.5% in 95% ethanol, Sigma) for 2 minutes. The stained sections were examined under a bright-field microscope (Nikon Eclipse Ni-E) equipped with a 20× objective lens (NA 0.75) and a DS-Fi3 camera. Images were analyzed with NIS-Elements AR 5.21 software to assess tissue morphology, inflammatory response, and new bone formation.

#### Immunofluorescence staining of type 1 collagen (Col1)

As described above, sections from each group were randomly selected and dewaxed. Antigen retrieval was performed by heating the sections in citrate buffer (10 mM, pH 6.0; Sigma) at 95 °C using a Biocare Medical system for 20 minutes. The slides were cooled to room temperature, washed with PBS (3 × 5 minutes), and blocked with 5% normal goat serum (Vector Laboratories) in PBS containing 0.3% Triton X-100 (PBST) for 1 hour at room temperature. For primary staining, the sections were incubated overnight at 4 °C in a humidified chamber with a Col1 antibody (ab21286, Abcam; 1:200) diluted in 1% BSA/PBST. After washing with PBST (3 × 5 minutes), slides were incubated with an Alexa Fluor 488-conjugated secondary antibody (Invitrogen; 1:500) for 2 hours at room temperature in the dark. Nuclei were counterstained with DAPI (1 μg/mL, Invitrogen D1306) for 5 minutes, followed by three PBS washes (5 minutes each). Finally, the sections were mounted using ProLong Gold Antifade Mountant (Invitrogen) and imaged with a confocal microscope (Zeiss LSM 880 Airyscan). The fluorescence intensity of Col1 was evaluated to assess ECM deposition.

### Quantification and statistical analysis

The sample size of each statistical analysis is n ≥ 3. All data are expressed as mean standard deviation. One way ANOVA and t-test were used to evaluate the differences between the experimental groups. SPSS software version 26.0 (SPSS Inc., Chicago, USA) was used for statistical analysis. A *P* value < 0.05 indicated statistical significance.
